# Identification and characterisation of novel potential phospho-acceptor sites in HPV-16 E7

**DOI:** 10.1016/j.tvr.2023.200270

**Published:** 2023-09-01

**Authors:** Oscar Trejo-Cerro, Justyna Broniarczyk, Nezka Kavcic, Michael Myers, Lawrence Banks

**Affiliations:** a International Centre for Genetic Engineering and Biotechnology, Padriciano 99, I-34149, Trieste, Italy; b Department of Molecular Virology, Adam Mickiewicz University, Uniwersytetu Poznanskiego 6, 61-614, Poznan, Poland

**Keywords:** HPV E7, Phosphorylation, Cell transformation

## Abstract

Several studies have described functional regulation of high-risk human papillomaviruses (HPVs), E6 and E7 oncoproteins via posttranslational modifications (PTMs). However, how these PTMs modulate the activity of E6 and E7, particularly in their targeting of cellular proteins, is not completely understood. In this study, we show that HPV16 E7 can be phosphorylated by casein kinase I (CKI) and glycogen synthase kinase 3 (GSK3). This principal phosphorylation occurs at threonine residues 5 and 7 with a more minor role for residues 19–20 in the N-terminal region of 16 E7. Intriguingly, whilst mutational analyses suggest that residues 5 and 7 may be dispensable for the transformation of primary baby rat kidney cells by E7, intact residues 19 and 20 are required. Furthermore, negative charges at these residues (TT19-20DD) enhance the pRb-E7 interaction and cells display increased proliferation and invasion capacities. Using a proteomic approach with a phosphorylated peptide spanning the TT19-20 region of HPV16 E7, we have identified a panel of new, phospho-specific E7 interacting partners. These results shed new light on the complexity of N-terminal phosphorylation of E7 and how this can contribute towards expanding the repertoire of E7 targeted pathways.

## 1 Introduction

Human Papillomaviruses (HPV) infections are mainly associated with cutaneous and mucosal epithelium. In some cases, persistent HPV infection may lead to carcinogenesis [1]. This carcinogenic process and the maintenance of the malignant phenotype is mainly associated with the expression of two viral oncoproteins: E6 and E7, which target the p53 and pRb tumour suppressors, respectively [2]. In addition, proteomic analyses have shown that E6 and E7 can interact with several targets [3,4], however the biological significance of these interactions and how they are regulated are still not fully understood [5].

Post-translational modifications (PTMs) occur after the translation of mRNA and can potentially change the stability, activity, and physical and chemical properties of proteins. In HPV infection, it has been shown that viral proteins are subjected to PTMs, which are important, both for the viral life cycle and during HPV-induced cancer [6]. In the case of E7, several PTMs have been described, including phosphorylation, ubiquitination and polyamination [6]. The HPV E7 N-terminal region contains the receptor motif for phosphorylation by CKII [7]. This motif is conserved between different HPV E7 types, as well as in the SV40 T and Ad E1A proteins from Simian Virus 40 and Adenovirus, respectively. In addition, a recent study has described an HPV16 E7 variant with an additional serine that can also be phosphorylated [8]. HPV E7 mutated at this CKII site shows a reduced ability to induce cell transformation and to maintain the transformed phenotype [7,9,10]. Moreover, in the context of the HPV genome, mutation of this phosphorylation motif results in reduced viral progeny production in raft cultures [11]. Biologically, CKII phosphorylation of HPV E7 seems to be required for optimal interaction with, and degradation of, pRb family members [[12], [13], [14]], Similarly, interactions of E7 with F-actin and with the TATA Box Binding Protein (TBP) are also CKII phosphorylation-dependent [15,16].

Phosphorylation of HPV16 E7 seems to be dependent on the cell cycle, occurring mainly at the CKII site during G1 phase, and progressively decreasing as the cells progress towards S-phase [17,18]. Interestingly, during S-phase the E7 serine 71 (S71) residue seems to be phosphorylated by a still unidentified kinase [17]. In addition, HPV16 E7 can be phosphorylated by dual-specificity tyrosine phosphorylation-regulated kinase 1A (DYRK1A) at threonine residues 5 and 7 (T5 and T7) [19] whilst T7 in the low-risk HPV6 E7 is phosphorylated by PKC [20], suggesting differential patterns of phosphorylation in different HPV types.

HPV E7 phosphorylation is required for viral infection and for viral oncogenic activity, and is also required for maintaining E7's ability to interact with several cellular targets [6]. Although several studies have focused on the main E7 phosphorylation motif, the CKII site, little is known about the role of other phosphorylation sites on E7: how they are regulated during infection and malignancy and whether these PTMs facilitate interaction with certain cellular targets. The aim of this study was to study new possible phospho-acceptor sites on E7 and their importance during its transforming activity and interaction with novel cellular targets. Using *in silico* and *in vitro* phosphorylation assays, we describe novel CKI and GSK3-mediated phosphorylation events at HPV16 E7 N-terminal threonine residues (T5 and T7, and to a much lesser extent at T19 and T20). Negative charges at TT19-20 residues of E7 seem to be required for an efficient E7-pRb interaction and promote higher cell proliferation and migration in HPV16 E7-expressing cells. Furthermore, using a proteomic approach, we observed that phosphorylated TT19-20 of E7 seems to provide a docking site for novel E7 interacting partners. Taken together these studies reveal a complex pattern of E7 phosphorylation within CR1 and CR2 which most likely affects target selection and cell transforming activity.

## 2 Material and methods

### 2.1 Cell culture

HEK293, primary baby rat kidney (BRK), H1299 cells as well as SiHa and C33A cervical cell lines were grown in Dulbecco's modified Eagle's medium (DMEM) containing 10% fetal bovine serum (FBS), glutamine (300 μg/ml) and penicillin-streptomycin (100U/ml) and incubated at 37 °C with 10% CO_2_.

### 2.2 Plasmids and transfections

The C-terminal FLAG/HA-tagged pCMV HPV16 E7 was kindly donated by Karl Münger [21]. The pJ4ΩHPV-16 E7 and EJ-ras plasmids have been previously described [22], as have the GST-tagged pGEX HPV16 E7 wild type and mutants [23]. In the case of pJ4ΩHPV-16 E7, the GST-tagged HPV16 E7, and FLAG/HA-tagged pCMV HPV16 E7 TT5-7AA, TT19-20AA, TT19-20DD mutants, they were generated by using Gene Art Site-Directed mutagenesis kit (Invitrogen) using the following primers: TT5-7AA forward primer, 5′ GAC CAT GCA TGG AGA TGC ACC TGC ATT GCA TGA ATA TAT-3′ and TT5-7AA reverse primer, 5′-ATA TAT TCA TGC AAT GCA GGT GCA TCT CCA TGC ATG GTC-3'; TT19-20AA forward primer, 5′- GAT TTG CAA CCA GAG GCA GCT GAT CTC TAC TGT TAT-3′ and TT19-20AA reverse primer, 5′- ATA ACA GTA GAG ATC AGC TGC CTC TGG TTG CAA ATC-3'; TT19-20DD forward primer, 5′- GAT TTG CAA CCA GAG GAC GAT GAT CTC TAC TGT TAT-3′ and TT19-20DD reverse primer, 5′- ATA ACA GTA GAG ATC ATC GTC CTC TGG TTG CAA ATC-3'. All constructs were sequence-verified using Sanger sequencing.

The APμ2-HA (Addgene No. 32752) and IQGAP1-Myc (Addgene No. 30118) plasmids were purchased from Addgene. The AP3M1 plasmid was from SinoBiological. The HA-tagged pRb expression construct was kindly provided by James DeCaprio and the PTPN14-V5 plasmid was kindly provided by Jianmin Zhang [24]. The PTPN13-HA and PARD3-Myc plasmids have been previously described [25,26]. Transient transfection assays were carried out using a standard calcium phosphate precipitation protocol.

### 2.3 *In vitro* phosphorylation and *in vitro* translation assays

Akt, PKA, PKC and DNAPK kinase assay systems were obtained from Promega. Cdc 2 from Sigma; and CKI and GSK3β kinases were from NEB. Purified GST-proteins were washed twice with a kinase buffer (25 mM Tris-HCl, pH 7.5; 10 mM MgCl2; 0.1% NP-40) and then incubated with the respective kinases in presence of 2.5 μCi radioactively-labelled ATP-[γ-32P], according to the manufacturer's protocol. For GST-pulldown assays, the GST fusion proteins were phosphorylated in presence of regular ATP. After extensive washes with the kinase buffer, samples were analysed by SDS-PAGE and autoradiography.

For *in vitro* translation, proteins were *in vitro* translated using the TNT kit (Promega) and radiolabelled with [S^35^]-methionine (PerkinElmer). The indicated GST-fusion proteins were incubated with the *in vitro*-translated proteins for 2 h at 4 °C. Beads were then washed with lysis buffer and samples were analysed by SDS-PAGE and autoradiography.

### 2.4 GST pulldown and immunoprecipitation assays

In the case of GST pulldown assays, cells were transfected with the indicated plasmids, then were lysed with lysis buffer (50 mM HEPES pH 7.4, 150 mM NaCl, 1 mM MgCl2, 1% Triton X-100), supplemented with protease inhibitor cocktail I (Calbiochem). Cellular extracts were incubated for 3 h at 4 °C with the indicated GST fusion proteins bound to glutathione resin (Sigma-Aldrich) and then the bound proteins were assessed by Western blot.

For immunoprecipitation assays, H1299 or HEK293 cells were transfected with the indicated plasmids and after 48 h, cells were lysed with lysis buffer supplemented with proteose inhibitor cocktail and incubated with HA- or FLAG-agarose beads (Sigma-Aldrich) for 3 h on a rotating wheel at 4 °C. Beads were washed three times with lysis buffer and samples were analysed by Western blot.

### 2.5 Transformation and clonogenic assays

BRK cells from 9-day-old Wistar rats were seeded at low confluence and transfected with EJ-ras plus pJ4ΩHPV-16 E7, either wild-type or mutants. Transformed cells were maintained under selection with G418 for 2 weeks and then colonies were fixed, stained with Giemsa and counted manually. In parallel, some transformed cells were kept it in culture for clonogenic and invasion assays.

For clonogenic assays, BRK and C33A cells were seeded at low confluence and, in the case of C33A, cells were transfected with pCMV HPV16 E7 wild-type or mutants or an empty vector. Then, colonies were selected with G418 and after 12–15 days, cells were fixed, stained and counted using countPHICS software for ImageJ [27].

### 2.6 Matrigel invasion chamber assay

Matrigel invasion chambers (Corning BioCoat Matrigel Invasion Chamber) were brought to room temperature, and chambers were rehydrated with DMEM for 2 h at 37 °C. Transformed BRK cells, at 2 × 10^4^ cells in 200 μl of DMEM medium without serum, were loaded in the upper chamber. In parallel, 750 μl DMEM supplemented with 10% FBS was added to the bottom chamber. After 24 h, the non-invading cells were removed using a cotton swab, and cells on the lower surface were fixed and stained with 0.5% Crystal Violet in 5% glutaraldehyde for 20 min. After exhaustive washes, invasive cells were visualized and counted using a transmitted light microscope at 20× magnification and analysed using GraphPad Prism software.

### 2.7 Peptide pulldown

Peptide pulldowns and their subsequent analyses were carried out as described previously [28]. Briefly, lyophilized biotinylated peptides (control peptide: Biotin-RRLQRTVEQR; 16 E7 19–20 peptide: Biotin-13-LDLQPETTDLYCYEQL -28; 16 E7 19–20 phospho-peptide: Biotin-13-LDLQPET^P^T^P^DLYCYEQL-28) were resuspended in lysis buffer and then bound to streptavidin-conjugated Sepharose beads (GE Healthcare). Afterwards, HaCat cell extracts, that were previously pre-cleared with empty streptavidin-conjugated beads, were incubated with the respective peptide-bound streptavidin beads. After several washes, bound proteins were assessed by Western blot or sent to the Proteomic facility at the International Centre for Genetic Engineering (ICGEB) (Trieste, Italy) for analysis by mass spectrometry.

### 2.8 Antibodies and western blot

Mouse anti-HPV16 E7, mouse anti-α-actinin and mouse anti-c-myc were from Santa Cruz Biotechnology; mouse anti-HA-peroxidase was from Sigma-Aldrich; mouse anti-phospho-threonine was from Cell Signalling Technology; rabbit anti-AP3M1 was from Abcam; mouse anti-V5 was from Life Technologies. Secondary anti-rabbit and anti-mouse HRP antibodies were obtained from Dako.

For Western blot, cells were harvested using Laemmli sample lysis buffer and denatured by boiling for 10 min. Proteins were then separated by SDS-PAGE and transferred to nitrocellulose membrane. Membranes were blocked in 5% non-fat dry milk in PBS/Tween-20 (0.1%) and incubated with the respective primary antibody followed by incubation with species-specific secondary antibodies. Immunoblots were developed using the western lightning ECL reagent (GE Healthcare). Results show a representative image and for the quantification, films were scanned, and band intensities were determined using ImageJ software.

### 2.9 Statistical analysis

All the experiments were performed at least three times (unless otherwise stated) and data shows the mean and standard deviation of the mean. The statistical significance was determined using the unpaired two-tailed Student's *t*-test with GraphPad Prism software.

## 3 Results

### 3.1 HPV16 E7 is phosphorylated by CKI and GSK3

**Fig. 1:**
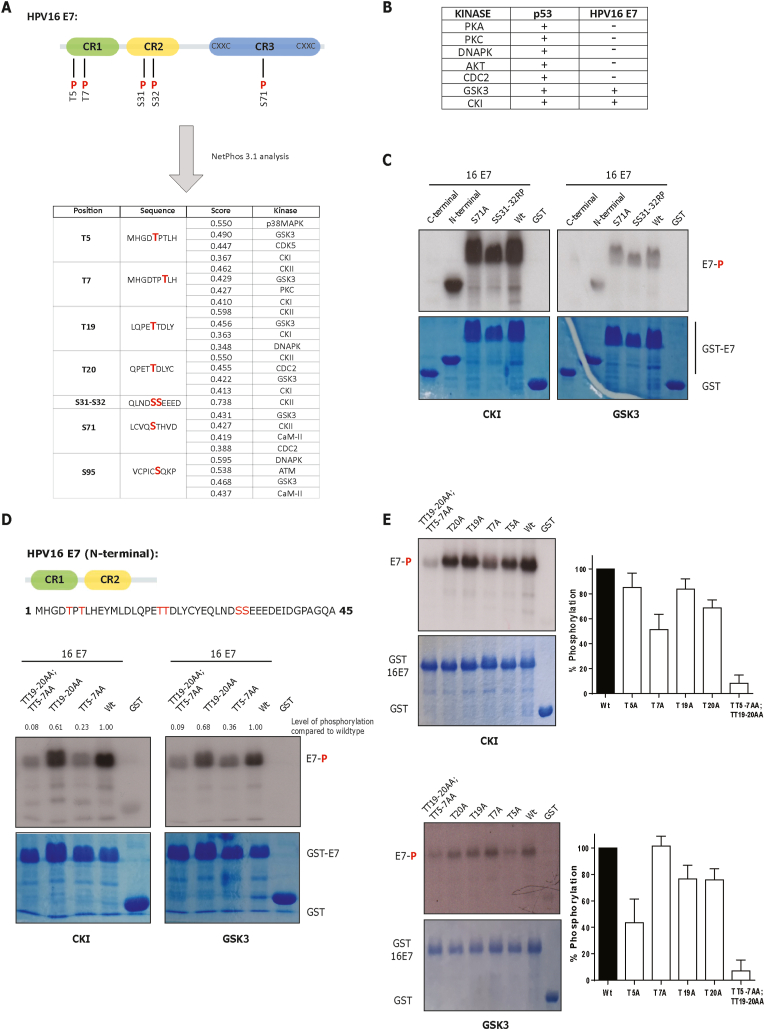
**CKI and GSK3 phosphorylate HPV16 E7 *in vitro*.** (A) *In silico* analysis of HPV16 E7 using NetPhos 3.1 server, which can predict serine, threonine or tyrosine potential phosphorylation sites. The cartoon of HPV16 E7 shows the previously reported phosphorylation sites. (B) A list of the kinases used in this study is shown. All the kinases tested phosphorylated p53, whilst only CKI and GSK3 could phosphorylate 16E7. (C-E) The indicated GST fusion proteins were incubated with the respective kinases in the presence of [γ-^32^P]-ATP. A representative autoradiogram for each kinase is shown, together with the Coomassie-stained SDS-PAGE gel for the indicated GST-protein.

HPV E7 is recognized as a serine phosphoprotein, with its main phosphorylation site for CKII at the N-terminal region in the CR2 domain. However, previous studies have described additional phosphorylation sites in HPV16 E7: threonines 5 and 7 are phosphorylated by DYRK1A kinase, and an S-phase-dependent phosphorylation at S71 by an, as yet unidentified, kinase has been reported [17,19] (Fig. 1A). In order to identify this kinase and other potential phospho-acceptor sites on HPV16 E7, we performed a *in silico* kinase analysis using the Netphos 3.1 server software [29]. The analysis identified CKII as a high-confidence candidate for the S31 and S31 residues (CKII site), demonstrating the reliability of this software (Fig. 1A). Interestingly, we detected several candidates for phosphorylation of S71 and other potential sites, therefore we decided to perform *in vitro* phosphorylation assays to confirm this analysis. For this purpose, purified enzymes were incubated, in the presence of radioactively labelled ATP, with either HPV16 E7 GST-protein, GST alone as a negative control, or GST-p53 as positive control. Intriguingly, of several kinases tested, only CKI and GSK3 could phosphorylate the E7 protein (summarized in Fig. 1B and C).

Having shown that HPV16 E7 is phosphorylated by GSK3 and CKI *in vitro*, we next wanted to ascertain which residue(s) were involved. We first attempted to explore the possibility of an S31 and S32 phosphorylation, so we repeated the *in vitro* phosphorylation assay using an HPV16E7 SS31-32RP mutant, in which the phospho-acceptor sites are destroyed. Interestingly, we observed that GSK3 and CKI phosphorylated this E7 mutant similarly to the E7 wild type, suggesting that these residues are not the main phosphorylation site for CKI/GSK3 (Fig. 1C). Then, we examined whether E7 S71 could be phosphorylated by these kinases: the results in Fig. 1C show that the E7 S71A mutant protein was still phosphorylated. Since we could not discern the main phosphorylation sites, we decided to perform a new phosphorylation assay using two halves of HPV16 E7 - the N-terminal, and C-terminal regions. Interestingly, we observed that only the N-terminal region of HPV16 E7 was phosphorylated (Fig. 1C), suggesting that the phospho-acceptor for GSK3 and CKI was located in the CR1/CR2 region of E7.

### 3.2 Threonines 5 and 7 in HPV16 E7 are phosphorylated by CKI and GSK3

Examining the N-terminal region of HPV16 E7, we noted that only a few serine/threonine residues could act as phospho-acceptors: the threonines 5, 7, 19–20, and the main phosphorylation site for CKII (S31 and S32) (Fig. 1D). Having shown that S31 and S32 are not the main sites phosphorylated by CKI and GSK3, we decided to focus on the T5, T7 and TT19-20 residues. We generated GST-16E7 mutants in which we destroyed the potential phosphorylation sites: 16 E7 T5 and T7 to A5 and A7 (TT5-7AA), E7 TT19-20AA, and a double mutant E7 TT5-7AA, TT19-20AA; we then repeated the *in vitro* phosphorylation assays. As we can see in Fig. 1D, the E7 mutants showed different degrees of phosphorylation by CKI and GSK3. The threonines 5 and 7 seem to be the main phospho-acceptor sites for GSK3/CKI, although there is consistent weak contribution from TT19-20. In the case of the double mutant (TT5-7AA; TT19-20AA), we observed complete abolition of phosphorylation (Fig. 1D). To further dissect the specific residues that are phosphorylated by CKI/GSK3, we repeated the phosphorylation assays using single E7 mutants (T5A, T7A, T19A and T20A). Interestingly, the results in Fig. 1E show that CKI and GSK3 have different phosphorylation patterns: T7 appears to be the main phosphorylation site for CKI whilst T5 is mainly phosphorylated by GSK3. However, in both cases we still observe a minor contribution from T19 and T20. These data suggest that CKI and GSK3 primarily phosphorylate T5 and T7 respectively but can also weakly phosphorylate the T19-T20 residues of HPV16 E7.

### 3.3 TT19-20 residues are important for HPV16 E7-induced transformation and pRb binding

**Fig. 2:**
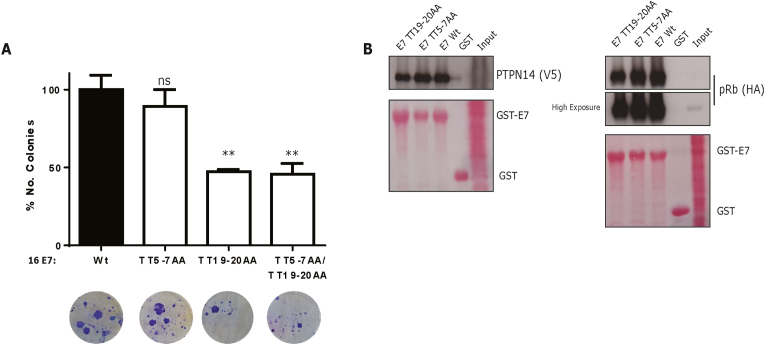
**E7-induced transformation is affected by mutations at threonine 19 and 20 in HPV16 E7.** (A) Primary BRK cells were transfected with plasmids expressing HPV16 E7 wild type or HPV16 E7 mutants (TT5-7AA, TT19-20AA, and the double mutant TT5-7AA; TT19-20AA), plus an EJ-ras expression plasmid. After 2 weeks' selection with G418, transformed cells were fixed, and colonies were stained and counted. A representative image is shown, and the number of colonies is expressed as the percentage of colonies obtained in cells transfected with HPV16 E7 Wt and EJ-ras, which was taken as 100%. (B) HEK293 cells were transfected with the indicated plasmids and cellular extracts were incubated with the indicated GST-protein and the bound proteins were determined by Western blot. The arithmetic means ± standard deviations are shown. **: p < 0.01; ns: not significant.

Having found that threonine residues in the N-terminal part of 16 E7 are potentially phosphorylated, we next wanted to determine the biological relevance of this. Therefore, we performed BRK-transformation assays using 16 E7 wild type and the respective E7 mutants in which these phosphorylation sites are destroyed (TT5-7AA, TT19-20AA and the double mutant TT5-7AA; TT19-20AA). Fig. 2A shows that mutations at threonine 5 and 7 do not affect E7-induced transformation, but that, interestingly, mutations at TT19-20 significantly reduce the number of transformed colonies (Fig. 2A). In agreement with this, the double E7 mutant (TT5-7AA; TT19-20AA) displayed a similar reduction of transformed colonies. E7-induced transformation is a complex and multifactorial process [5] and some E7 interactors such as pRb and PTPN14, are known to inhibit the E7 transforming activity [9,23]. Therefore, we asked whether the interactions of E7 with these tumour suppressors were affected by mutations at these E7 regions (TT5-7AA; TT19-20AA). Whilst E7 TT19-20AA showed a modest decrease in pRb binding, no changes were observed in PTPN14 interaction (Fig. 2B). In the same line, the TT5-7AA mutant showed no major changes in either pRb or PTPN14 binding. It is not surprising that PTPN14 interaction is unaffected as the main binding sites are located in the C-terminus of E7 [30]. All these data together suggest that threonines 5 and 7 are dispensable for transforming BRK cells, whilst the threonine 19 and 20 in HPV16 E7 are important for cellular transformation.

**Fig. 3:**
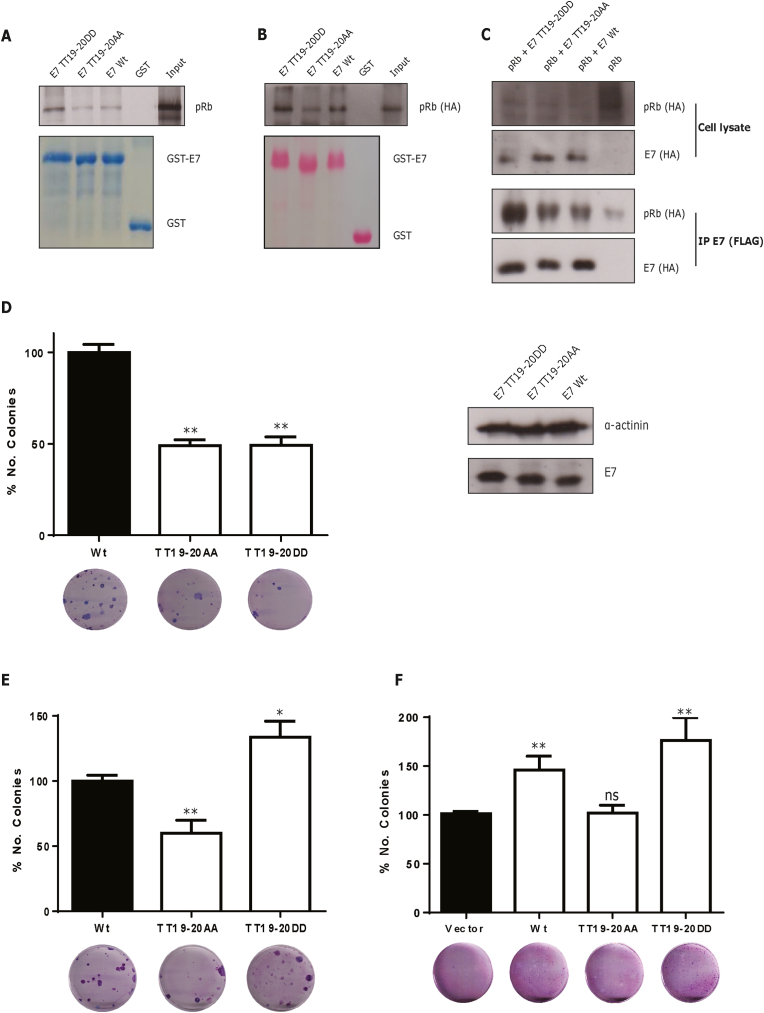
**Negative charges at threonine residues 19**–**20 of HPV16 E7 play an important role in pRb binding and cell proliferation.***In vitro* translated radioactively-labelled pRb (A), or cellular extracts from HEK293 cells transfected with a pRb-HA-expressing construct (B), were incubated with the indicated GST-protein and the bound proteins were assessed by Western blot. (C) HEK293 cells were transfected with pRb-HA and FLAG/HA-tagged HPV16 E7 wild type or mutant-expressing plasmids and co-immunoprecipitated with anti-FLAG agarose beads and analysed by Western blot. (D) A BRK cell transformation assay was carried out as described in Fig. 2, also including the HPV16 E7 wild type and E7 mutants (TT19-20AA and TT19-20DD). A representative immunoblot showing the expression of these E7s is shown. For colony formation assays, equal numbers of BRK cells previously transformed with the indicated 16 E7s (E) or C33A cells transfected with the respective constructs (F) were grown at low confluence and after 12–15 days, colonies were fixed, stained and counted. A representative image is shown, and the number of colonies is expressed as the percentage of colonies obtained using E7 wild-type (E) or in empty vector transfected-cells (F), which was taken as 100%. In all cases, the arithmetic means, ± standard deviations, from at least three independent experiments are shown. **: p < 0.01; *: p < 0.1; ns: not significant.

Whilst the threonines at 5 and 7 are clearly important phospho-acceptor sites, as reported here, and also shown previously (Liang et a. 2008), it is also apparent that TT19-20 can be recognized by different kinases, which appear to be biologically more relevant, at least in terms of cell transforming activity in BRK assays. We therefore decided to focus our attention on this region of E7 to ascertain how phosphorylation of TT19-20 might regulate E7 function. Earlier studies have reported that phosphorylation of E7 by CKII is relevant for E7-induced cell transformation [7,31]. In addition, an intact CKII phosphorylation site seems to be relevant for E7's targeting/inactivating pRb [14,27]. A crystal structure of pRb, bound to an HPV16 E7-peptide containing the LxCxE motif, suggests that negative charges downstream of this motif may facilitate the pRb-E7 binding [32]. Additionally, negative charges upstream of this region also seem to be important for the interaction [33]. In support of this, we observed a modest decrease in pRb interaction using the E7 TT19-20AA mutant (Fig. 2B). Hence, we hypothesized that residues adjacent to HPV16 E7 TT19-20 could impact upon pRb binding. To further investigate this, we performed GST-pulldown assays using HPV16 E7 wild type, or either the phospho-dead (TT19-20AA) or phospho-mimic (TT19-20DD) mutant GST-proteins. Radio-labelled, *in vitro* translated pRb (Fig. 3A), or cellular extracts from cells overexpressing pRb-HA (Fig. 3B), were incubated with the respective GST-proteins, and the binding of pRb was determined by autoradiography and Western blot, respectively. As expected, we observed that HPV16 E7 wild type interacts with pRb; more interestingly, the phospho-mimic E7 mutant appeared to interact more strongly than wild type with pRb. To confirm these *in vitro* results, we overexpressed HA-FLAG-tagged HPV16 E7 wild type or mutants (TT19-20AA and TT19-20DD), together with pRb-HA; and the cell extracts were immunoprecipitated using anti-FLAG beads. As we can observe in Fig. 3C, more pRb was immunoprecipitated with the phospho-mimic HPV16 E7 mutant than with wild type or E7 TT19-20AA. Taken together, the above results suggest that negative charges upstream of the LxCxE motif in HPV16 E7 enhance E7-pRb interaction above the binding observed for E7 wild type.

Our previous data suggests that threonines 19 and 20 are important for HPV16 E7-induced transformation (Fig. 2A). To investigate whether negative charges at these positions are relevant for cell transformation, we decided to repeat the BRK assays, but this time we included the E7 phospho-mimic mutant. Strikingly, we observed that both the phospho-dead and the phospho-mimic E7s were defective in transforming BRK cells (Fig. 3D). We monitored the expression of E7 proteins to verify that the mutations at these positions did not affect the stability of E7. Fig. 3D shows that all the E7s tested were expressed comparably, showing that the stability was unaffected and suggesting that intact threonine 19 and 20 residues in HPV16 E7 are crucial for the capacity of this oncoprotein to induce cell transformation in BRK cells.

### 3.4 Negative charges upstream of LxCxE motif are relevant for E7-induced cell proliferation

Although the BRK assays suggested that negative charges at threonine residues 19 and 20 in HPV16 E7 adversely affect E7-induced transformation, our pulldown assays clearly show that the E7 with phospho-mimic mutations at these residues is more efficient in interacting with pRb protein (Fig. 3A–C). Since the main function of pRb lies in its ability to regulate cell cycle progression, we decided to explore the role of E7 TT19-20 phosphorylation in the context of cell proliferation. For this purpose, we decided to generate stable BRK cell lines using E7 wild type or the phospho-dead or phospho-mimic mutants, respectively. Although only a reduced number of BRK colonies are obtained using E7 19–20 mutants, we still can get transformed cells. Hence, we repeated the BRK transformation assay using the plasmids expressing HPV16-E7 wild type, E7 TT19-20AA or E7 TT-19-20DD to generate a polyclonal population of transformed BRK cells that stably express the respective E7 proteins. Later, we collected the transformed cells and seeded equal numbers of cells at low confluence, and then evaluated their ability to form colonies (clonogenic assay). This assay allows us to determine, once the BRK primary cells are transformed, whether phosphorylation at this E7 region is relevant during the promotion of cell growth by this oncoprotein. Interestingly, the E7 phospho-dead mutant-expressing cells produced fewer colonies than the E7 wild type cells (Fig. 3E). In contrast, the E7 phospho-mimic mutant produced a slight but significant increase in the number of colonies compared with the wild type. To determine whether this phenotype was specific only for BRK cells, or was common to other cell lines, we decided to perform a further colony formation assay in C33A cells. Cells were transfected with either an empty vector or HPV16 E7 wild type or mutants (TT19-20AA and TT19-20DD); after 2 weeks’ selection, colonies were fixed, stained and counted. As we can see in Fig. 3F, we obtained similar results to the BRK clonogenic assay, in which the E7 phospho-mimic mutant produced the highest number of colonies, followed by E7 wild type, then the E7 phospho-dead mutant. These results indicate that negative charges at 19–20 position in HPV16 E7 are relevant for inducing cell proliferation, most likely through the association with pRb.

**Fig. 4:**
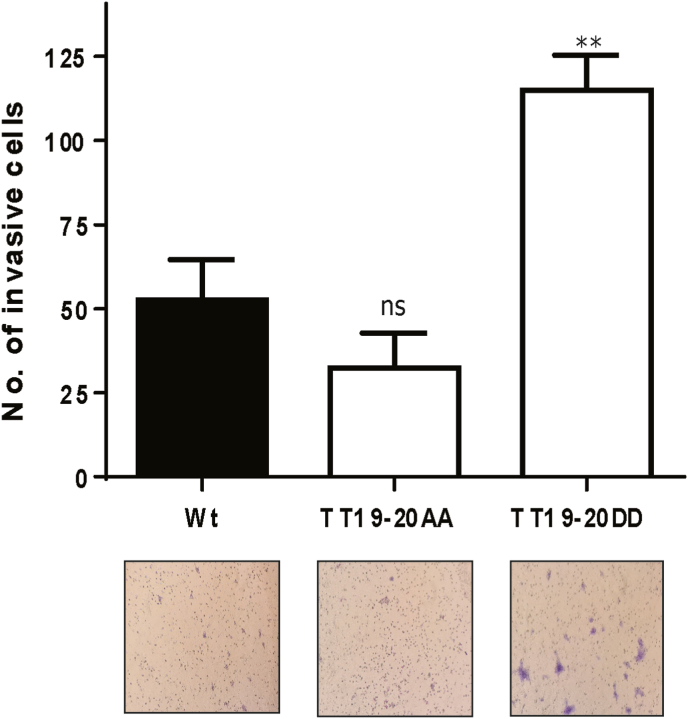
**The threonine 19**–**20 residues in HPV16 E7 are important for the invasive capacity of transformed cells.** Transformed BRK cells expressing the indicated HPV16 E7 constructs were seeded in the upper part of matrigel invasion chambers in serum-free medium, and DMEM with serum (10%) was used as chemoattractant in the lower chamber. After 24 h, invasive cells were fixed and stained. A representative image is shown, and the quantification of the mean number of invasive cells from at least three independent experiments. The arithmetic means, ± standard deviations, are shown. **: p < 0.01; ns: not significant.

Having shown that phospho-mimic mutations of HPV16 E7 threonine residues 19–20 can positively affect E7-induced cell proliferation, we wondered whether this region might be involved in the invasive capacity of transformed cells. To explore this, we performed a Matrigel invasion assay using the E7-transformed BRK cells. Interestingly, we found that cells transformed with the E7 phospho-mimic mutant were most invasive, compared with those transformed with the E7 wild type and the E7 phospho-dead mutant (Fig. 4), suggesting that the novel TT19-20 phospho-acceptor site is relevant for the ability of transformed cells to invade through a collagen matrix.

### 3.5 Proteomic analysis reveals novel phospho-specific interactors for 16 E7 TT19-20 region

**Fig. 5:**
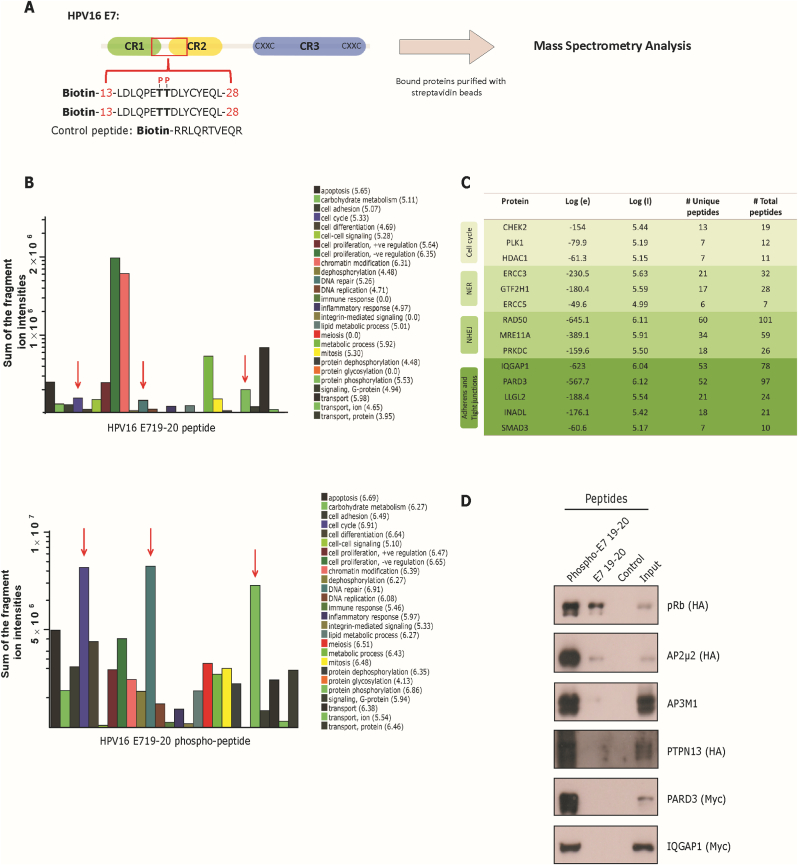
**Protein interaction analysis using a HPV16 E7 phospho- and non-phospho-peptide.** (A) Biotinylated HPV16 E719-20 phosphorylated, and non-phosphorylated peptides and a control peptide were conjugated with streptavidin magnetic beads and incubated with HaCat cellular extracts. The beads were recovered, and bound proteins were identified by mass spectrometry. (B) Protein analysis of the cellular processes enriched in each HPV16 E7 peptide pulldown. The enrichment was calculated using Log (I), which is indicated next to each cell process in parentheses. The red arrows highlight relevant pathways where E7 is involved. (C) A list of cellular pathways with proteins recovered only with the HPV16 E719-20 phospho-peptide is shown. Log (e): the base −10 log of the expectation that the assignment is stochastic; Log (I): the base −10 log of the sum of the intensities of the fragment ion spectra; the number (#) of unique peptide sequences and total number of tandem mass spectra that can be assigned to each protein. NER: Nucleotide excision repair; NHEJ: Non-homologous end-joining. (D) HEK293 cells were transfected with the indicated plasmids and cellular extracts were incubated with the respective peptides and bound proteins were detected by Western blot.

Phosphorylation of proteins can promote conformational changes or generate a docking site for interaction partners and, indeed, this holds true in the case of E6 and E7 oncoproteins [6]. In order to search for novel cellular phospho-specific interactors for this E7 region, we designed phosphorylated and non-phosphorylated biotinylated peptides corresponding to the HPV16 E7 TT19-20 region (Fig. 5A). HaCat cellular extracts were then incubated with the respective peptides and bound cellular proteins were identified by mass spectrometry. Strikingly, we observed a substantial diversity in the proteins recovered from the phospho-peptide, versus the non-phosphorylated peptide (Supplementary Table 1) (Fig. 5B). Since both peptides contain the LxCxE motif, we expected to see the pRb protein pulled down, as a positive control. As expected, we could detect pRb in both conditions, indicating that the pulldown assays worked properly (Supplementary Table 1). Interestingly, the number of cellular proteins recovered with the HPV16 E719-20 phospho-peptide was ≈4 times higher than with the non-phosphorylated peptide, and this is reflected in the number of different cellular processes affected by proteins enriched by the phospho-peptide (Fig. 5B). Proteins involved in cellular pathways such as cell cycle, DNA repair and tight junctions were detected exclusively in the phospho-peptide pulldown (Fig. 5B–C). Additionally, we found a range of DEAD-like helicases (such as DDX60, SETX and DDX3X), Adaptor Complex proteins (such as AP2 and AP3 proteins) and PDZ proteins (such as PTPN13, PARD3, DLG5 and SCRIB). To validate some of the novel targets, we overexpressed some cellular proteins in HEK293 cells and cell extracts were incubated with the respective peptides. We also included pRb as a positive control, since both peptides encompass the LxCxE motif. The results in Fig. 5D show a specific interaction of the selected targets only with the HPV16 E719-20 phospho-peptide, confirming the mass spectrometry data. We detected a pRb interaction with both peptides, although the E7 phospho-peptide interaction was stronger. These results are in line with our previous data, where the negative charges flanking the LxCxE motif increase the pRb-E7 interaction. We have previously reported that HPV16 E7 contains an AP2 recognition motif overlapping with the pRb LxCxE recognition sequence (25-YEQL-28) [34]. Our peptides also include this motif and, accordingly, we could detect an interaction with the AP2-μ2 adaptor protein subunit (Supplementary Table 1 & Fig. 5D). Similar to the interaction with pRb, the E7-AP2 interaction seems to be enhanced by CKII phosphorylation [34] and, interestingly, we observed that phosphorylation of TT19-20 upstream of the AP2-binding motif is also important for this interaction.

**Fig. 6:**
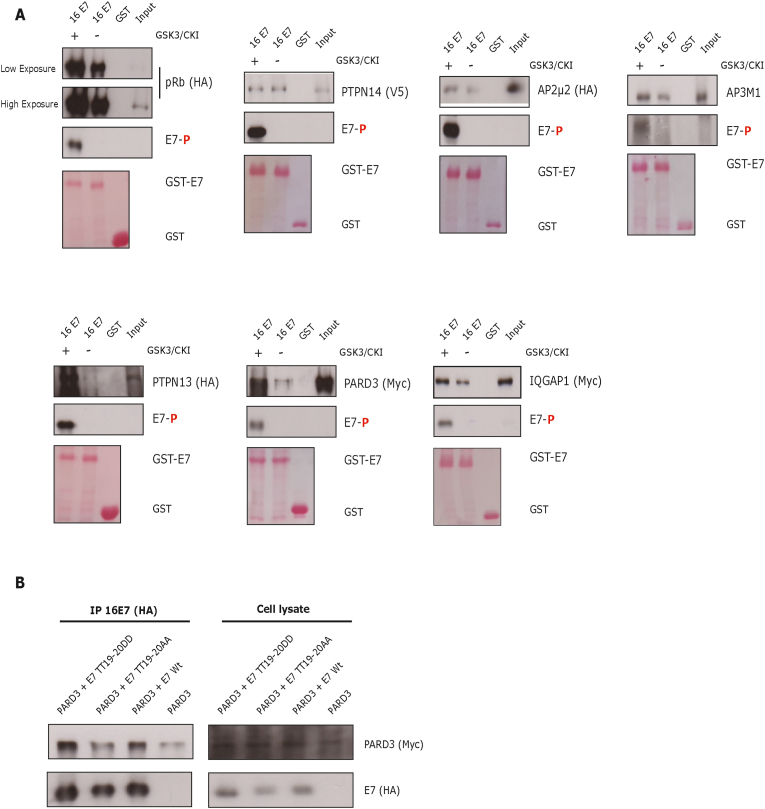
**Phosphorylation of HPV16 E7 by CKI/GSK3 is important for the interaction with specific targets.** (A) GST-E7 fusion protein or GST alone were phosphorylated with CKI and GSK3 kinases and then incubated with HEK293 cell lysates expressing the indicated constructs. Phosphorylation of E7 and interacting proteins were analysed by SDS-PAGE and Western blot. The lower panels show the Ponceau stain for the GST proteins. (B) H1299 cells were transfected with PARD3-myc and FLAG/HA-tagged HPV16 E7 wild type or mutant-expressing plasmids and co-immunoprecipitated with anti-HA agarose beads and analysed by Western blot.

To verify that these phospho-specific interactions occur in the context of the full-length HPV16 E7 protein, we performed E7 GST-pulldown assays. For this purpose, HPV16 E7 GST-protein was phosphorylated by CKI/GSK3 kinases and was then incubated with cellular extracts of HEK293 overexpressing some of the phospho-specific cellular targets. As negative control we included PTPN14 protein, which has been reported to interact with E7 through its C-terminal region [23,35], and as positive control we used pRb. When GST-E7 was preincubated with CKI/GSK3 we observed a higher interaction with pRb, whilst no difference was seen in the case of PTPN14 binding (Fig. 6A), as expected. In the case of the AP2 protein complex, it has been shown that phosphorylation of its cargoes is required for an efficient binding with the complex [34,36,37], and here, similarly, we observed a higher interaction with these proteins when E7 was phosphorylated. PTPN13 and PARD3 have been reported to be high-risk HPV E6 interactors [38,39], and our results show that phosphorylation by CKI/GSK3 promotes the interaction between GST-E7 and these PDZ domain-containing proteins (Fig. 6A). The IQ motif-containing GTPase-activating protein 1 (IQGAP1), a PI3K scaffold protein, is overexpressed in different types of cancers and moreover, it was found to be required during PI3K signaling induction by HPV16 oncoproteins [40]. Our data suggests that HPV16 E7 can interact with this cellular protein and, importantly, that CKI/GSK3 phosphorylation enhances this interaction.

To verify these *in vitro* results, we analysed the interaction of HPV16 E7 with PARD3 *in vivo*. For this purpose, we overexpressed HA-FLAG-tagged HPV16 E7 wild type or phospho-mutants together with PARD3-myc and cellular extracts were immunoprecipitated using anti-HA beads. As we can observe in Fig. 6B, the phospho-mimic HPV16 E7 mutant binds more efficiently to PARD3. All these data together highlight the importance of the novel CKI/GSK3 phospho-acceptor (TT19-20) in HPV16 E7, revealing novel potential phospho-specific interactors of this region.

## 4 Discussion

Early studies described HPV16 E7 as a serine phospho-protein, having a main phosphorylation site for CKII [41,42]. Somewhat later, other phosphorylation sites were described, including a phosphorylation in the CR1 region by DYRK1A and a phosphorylation at S71 in the C-terminal region of E7 by an unknown kinase [17,19]. Here, we describe new potential phosphorylation events for E7 proteins, reporting potential novel CKI/GSK3 phospho-acceptor sites mainly at threonines 5 and 7 and, to a lesser extent, at threonine residues 19–20 in HPV16 E7.

Previously, it had been shown that HPV16 E7 could be phosphorylated by DYRK1A at threonine residues 5 and 7, which extended the half-life of E7 [19]. Although we did not explore that possibility in this study, it is plausible that phosphorylation at the same location by CKI/GSK3 might produce the same phenotype. We were more interested in the novel phospho-acceptor site located at HPV16 E7 TT19-20, as that displayed the strongest phenotype in BRK cell transformation assays. Indeed, using C33A and BRK cells transformed with HPV16 E7 wild type and phospho-dead (TT19-20AA), or phospho-mimic (TT19-20DD) mutants in clonogenic assays, we clearly showed that negative charges are important for E7-induced proliferation. Since mutations at this site did not affect the stability of the oncoprotein, we hypothesized that HPV16 E7 phosphorylation positively affects cell proliferation. Mechanistically, we found that the HPV16 E7 19–20 phospho-mimic form binds more efficiently than wild type to pRb, and it has been previously proposed that an acidic patch downstream of the LxCxE motif is required for an efficient pRb interaction [32]. In accordance with this, positive charges flanking the LxCxE motif decrease the pRb binding affinity, whilst phosphorylation of E7 at the CKII site enhances the interaction with pRb protein [10,[12], [13], [14]]. Moreover, another study using peptides suggested that negative charges upstream of the LxCxE region are also important for an efficient binding [33]. In agreement with this, substitution of the negative residue aspartic acid in an HPV16 E7 peptide (21 position, 20-DLYCYEQL-29) for a positive residue (Arg, D21R) drastically reduced the affinity of pRb; conversely, substitution of glycine by aspartic acid just upstream of the pRb binding motif in the low-risk HPV6 E7 (G22D) substantially increased its binding affinity for pRb [13,43]. Here, we show that additional negative charges upstream of the LxCxE motif are also required for an efficient pRb interaction, suggesting that CKI/GSK3 phosphorylation could promote an optimal E7-pRb binding. This might also explain the high proliferation phenotype observed in cells transformed with the HPV16 E7 19–20 phosphomimic.

Since it has been shown that phosphorylation in both E6 and E7 oncoproteins can promote/disrupt the interaction with cellular targets [6], we sought to examine whether phosphorylation at HPV16 E7 TT19-20 could serve as a docking site for phospho-specific interactors. Strikingly, a proteomic analysis using non-phosphorylated and phosphorylated peptides encompassing the TT19-20 region of HPV16 E7, revealed two completely different profiles of binding proteins in the phospho- and non-phospho-peptides (Fig. 5). One caveat of this experiment is that we identified novel interactor partners using 16 E7 peptides, which may not necessarily reflect the structure/function of the full-length E7 oncoprotein. In addition, some cellular proteins might require different regions of E7 for an efficient interaction, which our peptide pulldowns would not detect. Therefore, it is crucial to validate the interactions of these novel cellular partners with the full-length E7 protein, as far as possible. Several of the proteins detected in our peptide pulldowns have already been described as E7 interacting partners, such as NBS1, HDAC1, RAD50 and CDK2 [[44], [45], [46]]. However, we also identified and confirmed by western blotting, several novel phospho-specific interacting partners, such as proteins from the AP2 complex and several proteins containing a PDZ domain. Interestingly, although various PDZ-containing proteins have been mainly associated with high-risk HPV E6 oncoproteins, some have also been observed in the context of E7. For instance, in the case of PARD3, an association with RhPV E7 oncoprotein has been described [25], nevertheless this interaction was associated with the PDZ-binding motif present on RhPV E7. Future studies will be focused on determining which regions of these PDZ domain-containing proteins and other targets identified in this work, are important for the phospho-interaction with E7. Additionally, recent studies showed a regulation of DLG1 levels by HPV18 E7 and, moreover, phosphorylation of E7 by CKII modulates the redistribution and stabilization of DLG1 and hScrib [47,48]. These data, together with our results presented here, suggest a possible interplay of E6 and E7 with certain cellular targets.

Obviously at this stage we can only speculate as to the identity of the principal kinases that might be responsible for phosphorylating E7 at TT19-20. The data presented show a potential role for CKI and GSK3, but their major phospho-acceptor sites lie at T5 and T7 respectively, and it seems unlikely that in an *in vivo* context the level of phosphorylation performed by these kinases on the TT19-20 phospho-acceptor site would be highly relevant. Nonetheless, the presence of acidic residues at this site, whether phospho-mimic or *bona fide* phosphate moieties introduced by CKI and GSK3 has a dramatic effect both upon E7 function and target pathway selection, and greatly enhances the functional flexibility of the E7 oncoprotein. Future studies will focus on investigating E7′s modulation of the newly identified target proteins, and in identifying other kinases potentially capable of targeting the TT19-20 phospho-acceptor site.

## Author statement

Oscar Trejo-Cerro: Conceptualization, Methodology, Validation, Formal analysis, Investigation, Visualization and Writing Original Draft; Justyna Broniarczyk: Validation, Investigation; Nezka Kavcic: Methodology, Investigation; Formal analysis; Mike Myers: Methodology, Data Curation, Formal analysis; Lawrence Banks: Conceptualization, Methodology, Resources, Writing Review & Editing, Supervision, Funding acquisition.

## Declaration of competing interest

The authors declare that they have no known competing financial interests or personal relationships that could have appeared to influence the work reported in this paper.

## Data Availability

Data will be made available on request.
